# Erectile dysfunction and the baculum

**DOI:** 10.1093/emph/eoz023

**Published:** 2019-08-26

**Authors:** Theodore C Smith, Laura Hechtel

**Affiliations:** 1 Medical Sciences, Indiana University School of Medicine, Bloomington, IN USA; 2 Department of Biology and Mathematics, D’Youville College, Buffalo, NY USA

## ERECTILE DYSFUNCTION

Erectile dysfunction (ED) is clinically defined as the inability to consistently acquire or maintain an erection of sufficient firmness for sexual intercourse. ED has several implicated causes with major predictors consisting of obesity, cardiovascular disease, medication use and psychological disease [1, 2]. Not just a condition of aging, ED has been shown to present in 38% of infertility patients [3]. Humans rely completely on increased blood flow to the penis to maintain an erection, while most primates receive additional assistance from the baculum or os penis.

The baculum is a bone located dorsally of the corpus spongiosum and varies in overall size, length and shape among the major groups of mammals possessing it [4].

## EVOLUTIONARY PERSPECTIVES

**Figure 1.**
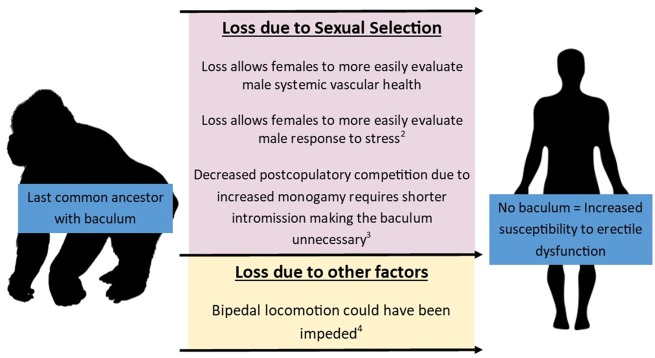
Possible hypotheses for loss of baculum in humans. (1) Hypothesis proposed by Richard Dawkins (1976) [7]. (2) Proposed by Zahavi and Zahavi (1997), further expounding on the Dawkins proposal [7]. (3) Proposed by Brindle and Opie (2016). (4) Proposed by Hsieh *et al.* (2012)

Humans are among the few primates who do not possess a baculum, along with tarsiers and several platyrrhines [5]. The distal ligament found within the glans penis in humans is analogous to the baculum in position and histological components and therefore implies an evolutionary relationship and function [6]. Highly susceptible to evolutionary forces, it is likely that the baculum has evolved 

independently 9 times and subsequently lost 10 times within mammal groups [4]. Several hypotheses have been put forth explaining the loss of the baculum in humans (Fig. 1). The first two hypotheses suggest a way for females to assess the overall physical and mental health of the male. The best supported hypothesis attributes increased monogamy in humans required more frequent mating of a shorter duration. In such a system, there would be no selective advantage to the baculum, and it could have been subsequently lost [5].

## FUTURE IMPLICATIONS

The evolutionary history of the human penis demonstrates the need for further investigation into the comorbid diseases already associated with ED [7]. As side effects and resistance to ED pharmaceuticals increase, a clinical perspective that utilizes how human evolutionary history has connected our reproductive health to our physical and mental health may be the key to better care. This evolution-informed clinical perspective should examine the upstream effects of ED as they may be a sign of more serious disease [7].
